# Serial changes in patient-reported outcome measures and satisfaction rate during long-term follow-up after total knee arthroplasty: a systematic review and meta-analysis

**DOI:** 10.1186/s43019-024-00241-6

**Published:** 2024-12-04

**Authors:** Jisu Park, Moon Jong Chang, Tae Woo Kim, Darryl D. D’Lima, Hyunkwon Kim, Hyuk-Soo Han

**Affiliations:** 1 Department of Orthopaedic Surgery, SMG-SNU Boramae Medical Center, Seoul National University College of Medicine, 20, Boramae 5th Road, Dongjak-Gu, Seoul, 07061 Republic of Korea; 2 Department of Orthopaedic Surgery, Seoul National University College of Medicine, Seoul, Republic of Korea; 3 Shiley Center for Orthopaedic Research and Education at Scripps Clinic, La Jolla, CA 92037 USA; 4 Department of Orthopaedic Surgery, Seoul National University Hospital, Seoul, Republic of Korea

**Keywords:** Total knee arthroplasty, Long-term follow-up, Patient-reported outcome measures, Satisfaction, Systematic review, Meta-analysis

## Abstract

**Purpose:**

This study aimed to investigate the sequential changes in patient-reported outcome measures (PROMs) and the satisfaction rate during long-term follow-up after total knee arthroplasty (TKA).

**Methods:**

Studies published until December 2023 were searched in MEDLINE, EMBASE, SCOPUS and Cochrane Library. The inclusion criteria were TKA as the primary procedure, a final post-operative follow-up period of at least seven years and reporting of PROMs data. The exclusion criteria were studies not reporting serial data of the same patient cohort, studies without mid-term data, comparative studies and reviews, comments or practice guidelines. Heterogeneity was assessed with the *I*^2^ and *tau*^2^ statistics. The quality of each study was evaluated using the methodological index for non-randomized studies (MINORS) criteria. The follow-up periods were divided into short-term, mid-term and long-term. Data were synthesised by narrative reviews and random-effects meta-analysis using standardised mean difference.

**Results:**

Among the 13 studies included in the review, six were included in the meta-analysis. The overall PROMs were maintained until the mid-term (0.14; 95% CI [confidence interval], −0.05 to 0.34; *I*^2^ = 96%; *tau*^2^ = 0.10; *P* = 0.16), but declined in the long-term (−0.23; 95% CI −0.34 to −0.13; *I*^2^ = 88%; *tau*^2^ = 0.04; *P* < 0.0001). According to the subgroup analysis, pain improved from the short-term to mid-term (0.21; 95% CI 0.14 to 0.29; *I*^2^ = 0%; *tau*^2^ = 0). Subscales including function (−0.28; 95% CI −0.52 to −0.03; *I*^2^ = 94%; *tau*^2^ = 0.09) and objective measure (−0.23; 95% CI −0.31 to −0.15; *I*^2^ = 62%; *tau*^2^ = 0.01) declined from the mid-term to long-term. The patient satisfaction rate remained consistent throughout the study period.

**Conclusions:**

The overall PROMs after TKA were maintained, with improvement observed in the pain subscale until the mid-term follow-up. However, in the long-term, overall PROMs, including function and objective measure, declined compared with those in the mid-term. Despite the decline in the physical aspects of PROMs over the long-term follow-up period, the patient satisfaction rate remained consistently high throughout the study period. Providing this information to patient pre-operatively may assist in establishing realistic expectations.

*Trial Registration* This research was registered at PROSPERO (registration number: CRD42024578579).

**Supplementary Information:**

The online version contains supplementary material available at 10.1186/s43019-024-00241-6.

## Introduction

Despite encouraging results on the long-term survival rates of total knee arthroplasty (TKA) [1], patients still have concerns regarding clinical outcomes. Studies on patients’ concerns before arthroplasty have indicated worries regarding potential complications, walking ability and residual pain [2, 3]. In response to these concerns, patient experience has become crucial in evaluating TKA outcomes. Patient-reported outcome measures (PROMs) are the most widely used tool for this purpose. A growing body of literature has been published on PROMs, and efforts have been made to identify predictive factors for poor PROMs after TKA [4–8]. However, few studies have reported long-term follow-up data on PROMs after TKA, with most available literature comparing only the final follow-up state with the pre-operative state [9]. Research on how PROMs change over time within the same patient cohort is limited.

PROMs usually consist of multiple subscales, including as function, pain and range of motion. As each subscale focusses on different aspects, the overall score and the scores for each subscale may change in different directions over time. Patients undergoing TKA are generally old and often have multiple comorbidities [10]. As patients age, comorbidities may worsen, leading to a decline in function [11]. However, pain is a subjective experience and may be less influenced by ageing. Examining how PROMs change over time according to each subscale can provide valuable insights to the condition of TKA patients. Sharing this information during pre-operative counselling can help manage patient expectations and improve patient satisfaction with surgical outcomes.

Patient satisfaction is also an important factor when evaluating outcomes. There is often a discrepancy in patient and surgeon satisfaction due to differing levels of expectations [12–14]. When patients state they are ‘satisfied’, they may not be referring to the functional outcome after surgery, and the surgeon’s interpretation may differ from the patient’s intent. Although some studies have used PROMs as measures of patient satisfaction, PROMs typically provide scores for specific items that may not necessarily align with overall patient satisfaction [15, 16]. Therefore, it is important to directly assess how satisfied patients are with their results, rather than relying on other measures. However, similar to research on PROMs, studies investigating changes in patient satisfaction rates over long-term follow-up periods are limited.

Therefore, this study aimed to investigate (1) changes in PROMs across each subscale from short-term to mid-term follow-up, (2) changes from mid-term to long-term follow-up and (3) variations in patient satisfaction rates over the long-term follow-up periods. The authors hypothesised that overall PROMs would be maintained from the short-term to mid-term follow-up but would decrease from the mid-term to long-term follow-up, and that these changes would vary by subscale. Additionally, we assumed that the patient satisfaction rate would not change throughout the study period.

## Methods

This meta-analysis was conducted in accordance with the Preferred Reporting Items for Systematic Reviews and Meta-Analyses (PRISMA) checklist [17]. As this was a review of existing literature, institutional review board approval was not necessary for this study.

### Search strategy

Two reviewers independently identified records by searching the database up to December 2023 including MEDLINE, EMBASE, SCOPUS and Cochrane Library. The searches were made using the following keywords: ‘total knee arthroplasty’, ‘long-term’, ‘patient outcome assessment’, ‘patient satisfaction’ and ‘functional outcome’. The detailed search strategy is provided in Appendix 1. The titles and abstracts were reviewed to exclude irrelevant studies before eligibility. During the literature review, the reference lists of the eligible studies were verified for further inclusion.

### Inclusion and exclusion criteria

We sought to analyse the sequential changes in PROMs and the satisfaction rate of patients who underwent TKA according to the follow-up periods. The study inclusion criteria were as follows: (1) TKA as the primary procedure; (2) a final post-operative follow-up period of at least seven years; and (3) reporting of PROMs data. The exclusion criteria were as follows: (1) studies not reporting serial data of the same patient cohort; (2) studies without mid-term data; (3) comparative studies; and (4) reviews, comments or practice guidelines.

### Data extraction and quality assessment

Two authors independently reviewed the full texts of the articles. To assess the study quality, sample size, study design and level of evidence were collected. Furthermore, the quality of each study was evaluated using the methodological index for non-randomized studies (MINORS) criteria [18]. Data on follow-up periods, PROMs and the patient satisfaction rate were collected. Data in other forms (median, interquartile range and mean with 95% confidence interval [CI]) were converted to mean and standard deviation on the basis of the previous guideline [19].

### Patient-reported outcome measures

In the field of TKA, Western Ontario and McMaster Universities Osteoarthritis Index (WOMAC), Knee Society Score (KSS), Oxford knee score (OKS), Knee injury and Osteoarthritis Outcome Score (KOOS), Knee Society Clinical Rating (KSCR) and Hospital for Special Surgery (HSS) score are widely used disease-specific PROMs. These scoring systems address questions specific to arthritis or knee diseases. These disease-specific measures were subdivided into three categories: (1) ‘function’ for assessing patients’ general functional status such as walking and climbing stairs; (2) ‘objective measure’ for addressing alignment, range of motion and stability; and (3) ‘pain’ for addressing the pain. For example, KSFS, function subscale of WOMAC and function subscale of KSCR were categorised as ‘function’. When multiple scoring systems were presented for the same subscale, the main scoring system emphasised in each study was used for the analysis. Additionally, a meta-analysis was conducted on disease-specific PROMs.

In addition to these disease-specific scoring systems, those addressing patients' general quality of life (QoL) have also been used to evaluate TKA outcomes. The 12-Item Short Form survey (SF-12) and 36-Item Short Form survey (SF-36) are frequently used tools in this area, and comprised of two components: physical and mental health.

### Patient satisfaction rate

The most commonly used method of reporting satisfaction is through a single question about overall satisfaction (e.g. ‘Are you satisfied with the results?’). The responses were usually assessed using a Likert scale, typically with four categories: very satisfied, satisfied, dissatisfied and very dissatisfied [16]. In case of using four-scale answers, the patient satisfaction rate was defined as the percentage of patients who reported being very satisfied or satisfied with the four outcome aspects.

### Synthesis of results

Follow-up periods were divided into three groups: short-term, up to 1 year; mid-term, 2–5 years; and long-term, 7–10 years. This study analysed changes in PROMs over time within the same cohort. The changes of PROMs from short-term to mid-term and from mid-term to long-term were analysed. As the scoring system varied between the studies, the standardised mean difference (SMD) was used for comparison. If a scoring system was in the opposite direction, it was multiplied by −1 so that higher scores represented better results. A random-effects model was used to estimate the SMD for continuous data across the studies. A subgroup analysis was performed according to the aforementioned subscales. A *P*-value of < 0.05 was considered significant for pooled effects. All statistical analyses were conducted using R (version 4.2.2) and RStudio (version 2023.03.1 + 446) with the meta package.

### Risk of bias

The risk of bias in each study was assessed by qualitative review on the basis of the study quality (Table 1). Publication bias was measured using the Egger’s regression test. The heterogeneity of the results across the studies was assessed using *I*^*2*^ and *tau*^*2*^ statistics.

**Table 1:** Characteristics of included studies

Study, year (surgery year)	Subjects number	Study design	Patients demographics	Disease-specific PROMs	General QoL	Satisfaction	Level of evidence	MINORS score
Sebastia-Forcada [23], 2023 (2009–2012)	309	RC	69.2 years Female 63.7% BMI 30.8 OA	KSFS KSKS WOMAC	NR	R (4-scale)	III	13
Wylde [28], 2021 (2006–2009)	266	PC	70 years Female 64% BMI 30 OA	WOMAC KOOS AKSS	UCLA	R (4-scale)	II	10
Baek [26], 2021 (2009)	395	RS	67.5 years Female 89% BMI 27.0 OA 98%, Post-traumatic 1.5%, RA 0.5%	KSKS KSFS	NR	NR	III	10
Woo [20], 2021 (2006)	120	RS	65 years Female 80.8% BMI 27.9 OA	KSKS KSFS OKS	SF-36	NR	III	15
Bajada [31], 2019 (2005–2007)	31	RS	72 years Female 67.7% BMI NR OA	OKS	NR	NR	III	12
Scott [32], 2019 (2006–2007)	426	PC	69 years Female 62.5% BMI NR OA 87.9%	OKS FJS	SF-12	R (4-scale)	II	12
Arikupurathu [24], 2019 (1997–2002)	308	RC	69.5 years Female 62% BMI 28.4 OA 90.1%, Inflammatory 8.8%, Post-traumatic 0.6%, ON 0.3%	KSS KSFS	NR	R (yes or no)	III	11
Jiang [21], 2017 (1999–2003)	2080	PC	71 years Female 56.4% BMI NR OA 95.1%, RA 4.9%	OKS	NR	NR	II	12
Williams [22], 2013 (1994–2008)	1266	PC	71.5 years Female 61.4% BMI 29.9 NR	OKS	NR	NR	II	11
Arthur [30], 2013 (1998–1999)	235	PC	66.5 years Female 50.7% BMI 30.5 OA 88%, RA 9%, Post-traumatic 3%	AKSS OKS	NR	NR	II	12
Meding [27], 2012 1975–1989)	128	RS	63.8 years Female 73% BMI NR OA 82%, RA 14%, ON 4%	HSS KSCR	UCLA	NR	III	12
Watanabe [29], 2004 (1990–1993)	54	PC	72.3 years (OA), 65.7 years (RA) Female 86% (OA), 100% (RA) BMI NR OA 83%, RA 17%	KSKS KSFS	NR	NR	II	9
Schrøder [25], 2001 (1984–1986)	112	PC	78 years (at F/U) Female NR BMI NR OA 83%, RA 17%	HSS	NR	R (4-scale)	II	10

*PROMs* patient-reported outcome measures, *QoL* quality of life, *MINORS* methodological index for non-randomized studies, *BMI* body mass index, *OA* osteoarthritis, *RA* rheumatoid arthritis, *KSS* Knee Society Score, *KSKS* Knee Society Knee Score, *KSFS* Knee Society Function Score, *OKS* Oxford knee score, *WOMAC* Western Ontario and McMaster Universities Osteoarthritis Index, *HSS* Hospital for Special Surgery score, *KSCR* Knee Society Clinical Rating, *FJS* Forgotten Joint Score, *KOOS* Knee injury and Osteoarthritis Outcome Score, *AKSS* American Knee Society Score, *SF-36/12* Short Form 36/12-item, *UCLA* University of California Los Angeles activity score, *RC* retrospective cohort, *RS* retrospective study, *PC* prospective cohort, *R* reported, *NR* not reported, *F/U* follow-up

## Results

### Study selection

**Fig. 1:**
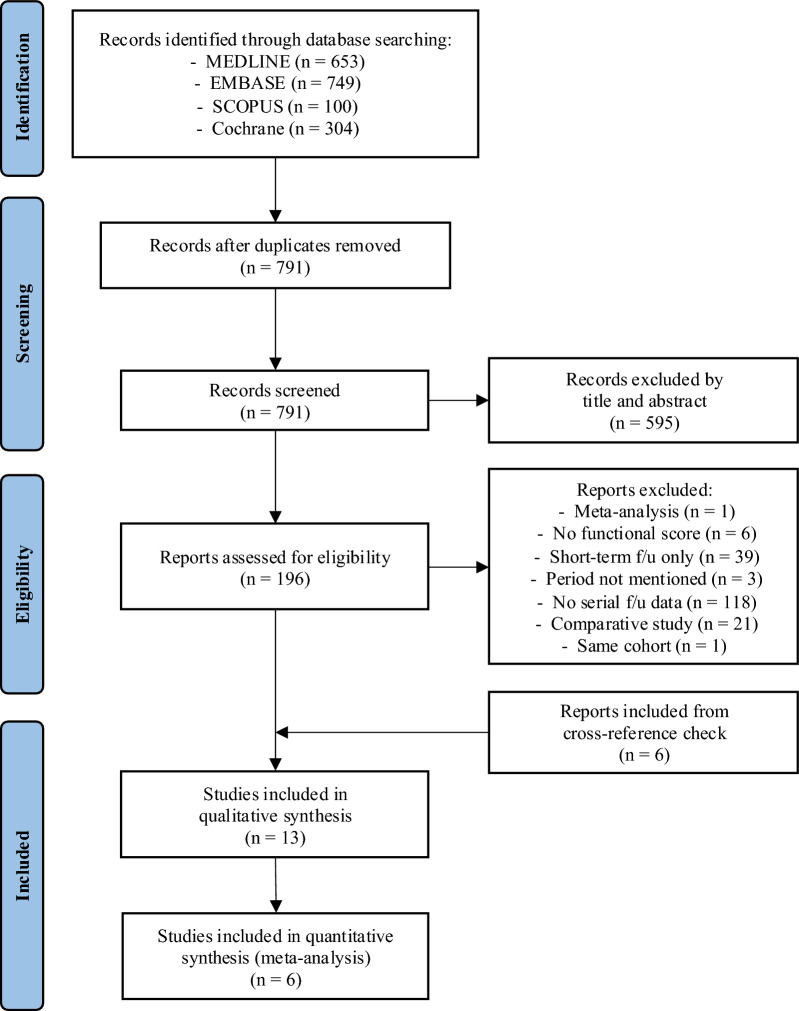
PRISMA flow diagram for study selection

The initial search resulted in 653 studies in MEDLINE, 749 in EMBASE, 100 in SCOPUS and 304 in Cochrane Library. After removing duplications, 791 studies remained, of which 196 were eligible for further investigation after exclusion on the basis of the title and abstract. After reviewing the eligible studies, 13 studies were selected for review, and six studies were included in the meta-analysis (Fig. 1).

### Study characteristics and quality assessment

The characteristics of the included studies are presented in Table 1. Primary TKA was performed in 5730 cases, with the number of cases per study ranging from 31 to 2080. All 13 studies reported disease-specific PROMs, with 11 reporting on function, seven on objective measures and eight on pain; three studies reported the serial general QoL outcomes. Patient satisfaction rates were reported in five studies.

According to the literature, studies with fewer than 270 cases were considered small [9]. Consequently, seven studies had insufficient sample sizes, potentially introducing bias; seven were prospective, and the remaining six were retrospective studies. MINORS scores ranged from 9 to 15 (Table 1, Appendix 2).

### Patient-reported outcome measures

**Fig. 2:**
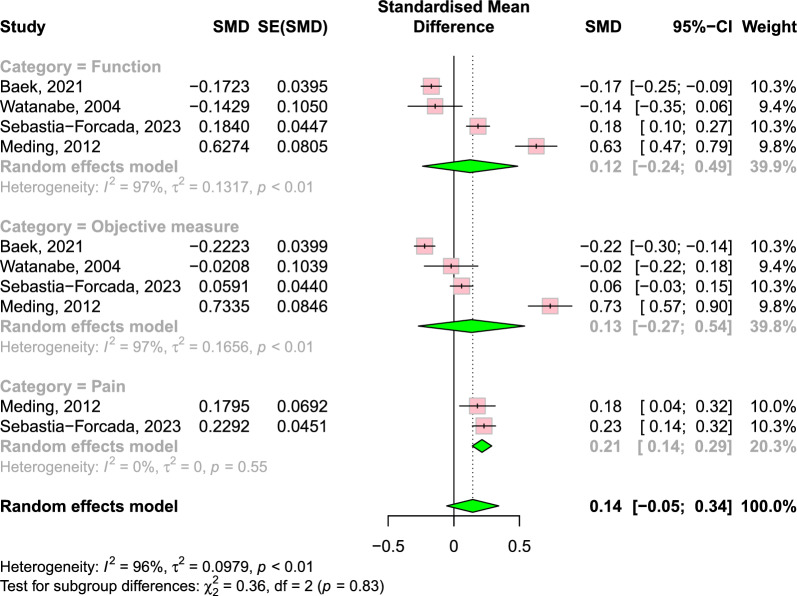
Forest plot of change of disease-specific PROMs from short-term to mid-term follow-up. Pooled-effects analysis demonstrated that overall disease-specific PROMs were maintained. However, in subgroup analysis, there was an improvement in pain score. SMD, standardised mean difference; SE, standard error; CI, confidence interval

**Fig. 3:**
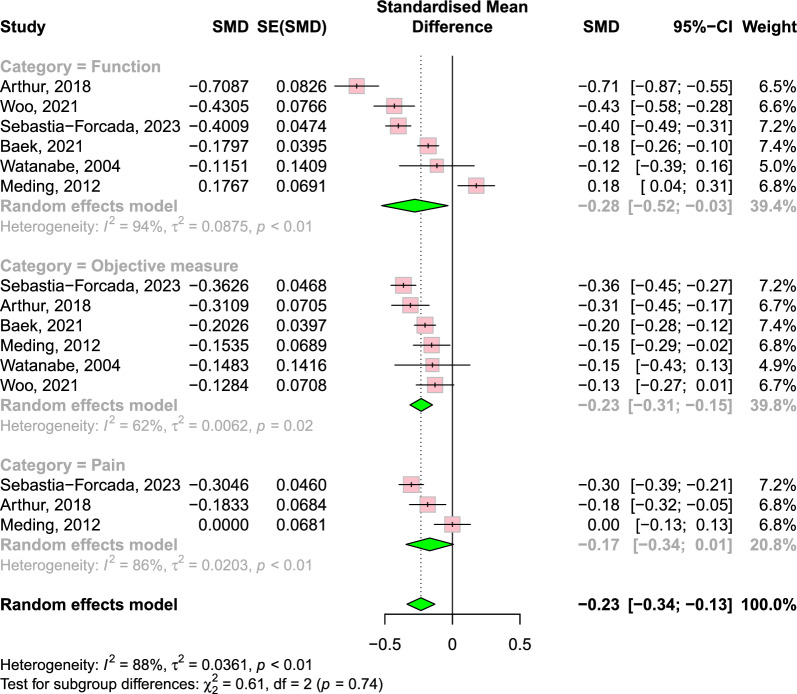
Forest plot of change of disease-specific PROMs from mid-term to long-term follow-up. The overall disease-specific PROMs declined. Also, scores in function and objective measure showed decline in subgroup analysis. SMD, standardised mean difference; SE, standard error; CI, confidence interval

The complete results are presented in Table 2. Outcomes were analysed using the following subscales: function, objective measure and pain for disease-specific PROMs; and general QoL. Pooled-effects analysis demonstrated that overall disease-specific PROMs were maintained from the short-term to mid-term (0.14; 95% CI −0.05 to 0.34; *P* = 0.16) (Fig. 2), but they declined from the mid-term to long-term (−0.23; 95% CI −0.34 to −0.13; *P* < 0.0001) (Fig. 3).

**Table 2:** Patient-reported outcome measures of included studies

Study, year (surgery year)	Reporting follow-up	Function	Objective measure	Pain	QoL
Sebastia-Forcada [23], 2023 (2009–2012)	3~6 months, 1, 3, 5, 7, 10 years	KSFS, WOMAC (Function): maximum score was achieved at 3 years, remain steady to 5 years, decreased from 5 years to 7 years, and remained steady to 10 years	KSKS: maximum score was achieved at 3 years, remain steady to 5 years, decreased from 5 years to 7 years, and remained steady to 10 years	WOMAC (Pain): maximum score was achieved at 3 years, remain steady to 5 years, decreased from 5 years to 7 years, and remained steady to 10 years	NR
V. Wylde [28], 2021 (2006–2009)	3 months, 1, 2, 3, 5, 7, 10 years	WOMAC (Function): increased until 1 year, remained steady until 10 years	NR	WOMAC (Pain): increased until 1 year, remained steady until 10 years	Patients reporting large improvement in their QoL ranged from 60% to 77% over the duration of follow-up UCLA: improved slightly at 3 months, and remained at this level for 10 years
Baek [26], 2021 (2009)	1, 3, 6, 10 years	KSFS: increased compared with pre-operative state, gradual subtle decline over time	KSKS: increased compared with pre-operative state, gradual subtle decline over time	NR	NR
Woo [20], 2021 (2006)	6 months, 2, 10 years	KSFS, OKS (Function): improved consistently until 2 years and decline from 2 to 10 years	KSKS: improve until 6 months and remain steady afterward	NR	PCS, MCS: improve until 6 months and remain steady afterward
Bajada [31], 2019 (2005–2007)	6 weeks, 6 months, 1, 2, 5, 10 years	NR	ROM: improved post-operatively and remained steady until 10 years	NR	NR
Scott [32], 2019 (2006–2007)	6 months, 1, 5, 10 years	NR	NR	NR	PCS: improved until 1 year and declined at 5 years MCS: did not change significantly over the study periods
Arikupurathu [24], 2019 (1997–2002)	1, 3, 5, 7, 10 years	KSFS: improved until 1 year, significant reduction at each subsequent visit	NR	KSS (Pain): improved until 1 year, no further improvement afterward	NR
Jiang [21], 2017 (1999–2003)	1~10 years annually	OKS (Function): average annual decrease of 0.2 points	NR	OKS (Pain): average annual decrease of 0.2 points	NR
Williams [22], 2013 (1994–2008)	1~10 years annually	OKS (Function): maximum scores were achieved within the first 2 years and decline gradually over time	NR	OKS (Pain): improved until 4 years and decline gradually over time	NR
Arthur [20], 2013 (1998–1999)	5, 10 years	AKSS (Function): declined from 80.5 at the mid-term to 68.9 at the long-term	AKSS (Knee): declined from 84.3 at the mid-term to 78.8 at the long-term	AKSS (Pain): declined from 44.3 at the mid-term to 41.3 at the long-term	NR
Meding [27], 2012 (1975–1989)	6 months, 1, 3, 5, 7, 10, 12, 15, 17, 20 years	HSS, KSCR (Function, Stair, Walking): diminished over 20-year follow-up, but until 10 years, remained steady	HSS, KSCR (Knee): did not diminish over time	HSS, KSCR (Pain): did not diminish over time	Not reported as serial data
Watanabe [29], 2004 (1990–1993)	1, 3, 5, 10 years	KSFS: remained significantly higher for 10 years after surgery compared with their pre-operative values	KSKS: remained significantly higher for 10 years after surgery compared with their pre-operative values	NR	NR
Schrøder [25], 2001 (1984–1986)	3, 5~7, 10 years	Walking distance: similar result of 10 year compared with 5–7 years, but inferior than 3 years	NR	Pain during walking decreased during 10 years	NR

*QoL* quality of life, *KSS* Knee Society Score, *KSKS* Knee Society Knee Score, *KSFS* Knee Society Function Score, *AKSS* American Knee Society Score, *OKS* Oxford knee score, *WOMAC* Western Ontario and McMaster Universities Osteoarthritis Index, *HSS* Hospital for Special Surgery score, *KSCR* Knee Society Clinical Rating, *PCS* physical component score, *MCS* mental component score, *UCLA* University of California Los Angeles activity score, *ROM* range of motion, *NR* not reported

### Function

In total, 11 studies reported on the functional subscales of PROMs. Among the ten studies that reported both short-term and mid-term data, seven studies reported a gradual decline in PROMs [20–26]. Two studies reported that it remained steady until 10 years post-operatively [27, 28]. The remaining one study did not report changes over time but showed that the functional aspect of PROMs remained higher than the pre-operative state at all times [29]. One study did not report short-term data and showed a decline from the mid-term to the long-term [30]. According to the meta-analysis, the functional part of PROMs showed no difference from the short-term to mid-term (0.12; 95% CI −0.24 to 0.49), but declined from the mid-term to long-term (−0.28; 95% CI −0.52 to −0.03).

### Objective measure

A total of seven studies reported on the objective measure subscale of PROMs; three reported that the objective measure of PROMs was maintained throughout the study periods [20, 27, 31], and the other two reported a subtle decline over time [23, 26]. One study did not report changes over time, but showed that the objective measure of PROMs remained higher than the pre-operative state [29]. The remaining one study did not report short-term data and showed a decline from the mid-term to long-term [30]. According to meta-analysis, the objective measure part of PROMs showed no difference from the short-term to mid-term (0.13; 95% CI −0.27 to 0.54), but declined from the mid-term to long-term (−0.23; 95% CI −0.31 to −0.15).

### Pain

A total of eight studies reported on the pain subscale of PROMs. Among the seven studies that had both short- and mid-term data, one reported gradual improvement [25], two reported improvement until the mid-term and decline afterward [22, 23], two studies reported improvement until the short-term and no change afterward [24, 28], one study reported no change over time [27] and one study reported a gradual decline over time [21]. One study did not report short-term data and showed a decline from the mid-term to long-term [30]. The meta-analysis showed an improvement in the pain score from the short-term to mid-term (0.21; 95% CI 0.14 to 0.29). The change from the mid-term to long-term did not reach statistical significance (−0.17; 95% CI −0.34 to 0.01).

### General quality of life

A total of three studies reported on general QoL after TKA. The mental part of the QoL remained steady over the study period in two studies [20, 32]. The physical part of the QoL remained steady throughout the study period in two studies [20, 28], but one study reported a decline at five post-operative years [32].

### Patient satisfaction rate

A total of five studies provided data on the patient satisfaction rate according to the follow-up period (Table 3). Among them, four studies provided patient satisfaction rate on the basis of the four-scale answers (e.g. very satisfied, satisfied, dissatisfied and very dissatisfied) [23, 25, 28, 32]. In their studies, the patient satisfaction rate was defined as the percentage of patients who reported being very satisfied or satisfied with the four outcome aspects. The remaining one study by Arikupurathu et al. reported patient satisfaction rate on the basis of two-scale, ‘yes or no’ answers [24]. In all studies, the satisfaction rate did not change substantially over the 10-year long-term follow-up period after TKA.

**Table 3:** Patient satisfaction rate of reported studies

	Study, year (surgery year)				
	Sebastia-Forcada [23], 2023 (2009–2012)	V. Wylde [28], 2021 (2006–2009)	Scott [32], 2019 (2006–2007)	Arikupurathu [24]^)^, 2019 (1997–2002)	Schrøder [25], 2001 (1984–1986)
Patient satisfaction rate	89.4% at 5 years, 87.1% at 7 years, 84.5% at 10 years showed satisfaction	91% at 1 year, 89% at 2 years, 85% at 3 years, 86% at 5 years, 90% at 7 years, 88% at 10 years showed satisfaction	88.3% at 1 year, 88.0% at 5 years, 88.4% at 10 years showed satisfaction	92.8% at 1 year, 96.2% at 3 years, 96.4% at 5 years, 97.1% at 7 years, 97.3% at 10 years showed satisfaction	89% at 3 years, 89% at 5–7 years, 92% at 10 years showed satisfaction

### Risk of bias across the studies

The heterogeneities among the studies were high, as reflected by *I*^2^ and *tau*^2^: 96% and 0.10 for the short-term to mid-term, respectively; and 88% and 0.04 for the mid-term to long-term, respectively. In the subgroup analysis, the *I*^2^ ranged from 0% to 97% and *tau*^2^ ranged from 0 to 0.17 for the short-term to mid-term, while *I*^2^ ranged from 62% to 94% and *tau*^2^ ranged from 0.01 to 0.09 for the mid-term to long-term. The Egger’s regression test showed no significant publication bias across the studies: *P* = 0.10 for the short-term to mid-term and *P* = 0.72 for the mid-term to long-term.

## Discussion

Historically, the primary emphasis in assessing long-term TKA outcomes has been implant survival. However, there has been an increasing focus on PROMs and patient satisfaction. The PROMs and patient satisfaction after TKA have been reported to improve compared with those in the pre-operative state, but few studies have focussed on their sequential changes over time [9]. This study aimed to evaluate the changes in PROMs and patient satisfaction rates over long-term follow-up periods after TKA. The main findings of this study were as follows: (1) overall PROMs after TKA were maintained, while the pain subscale improved from the short-term to mid-term; (2) overall PROMs, including function and objective measure, declined from the mid-term to long-term; and (3) the proportion of patients satisfied with the results remained consistent throughout the long-term follow-up period after TKA.

While the overall PROMs remained constant until mid-term, improvement was observed in the pain subscale. Among seven studies reporting changes in the pain subscale of PROMs from the short-term to mid-term, only one study noted a decline, with an average annual decrease of 0.2 points [21]. The remaining studies reported improvement or maintenance of the pain subscale scores until mid-term follow-up. Williams et al. reported that maximum OKS night pain score was measured at 4 years post-operatively [22]. Sebastia-Forcada et al. also reported a maximum WOMAC pain score (modified so that higher scores indicate better outcomes) at 3 years post-operatively [23]. Other aspects of PROMs, such as function, objective measure and QoL, did not exhibit differences between the short-term and mid-term. These findings imply that there is room for further improvement in pain, even 1 year after TKA. Among the various expectations patients have before undergoing TKA, pain relief is one of the most important goals that patients desire to achieve [33–35]. Additionally, post-operative residual pain was identified as a key predictor of patient dissatisfaction [36, 37]. DeFrance et al. conducted a systematic review and reported that most dissatisfaction after TKA was due to complications, unmet expectations, persistent pain and stiffness [38]. Therefore, when counselling patients during the pre-operative phase, it may be beneficial to inform them that post-operative pain can continue to improve beyond the short-term follow-up. This can help establish realistic expectations for patients, and setting an appropriate level of expectation for patients can enhance post-operative satisfaction [39].

When follow-up periods were extended to the long-term, overall PROMs, including function and objective measure, declined compared with those in the mid-term. Interestingly, a decrease was observed mainly in the physical aspect. This trend may be attributed to the decreased physical capabilities of ageing patients. As individuals age, muscle mass decreases by 0.5–1% per year, resulting in reduced strength and a decline in rapid force production [40, 41]. Sarcopenia in the elderly is a critical condition that results in decreased mobility, increased risk of falling and increased mortality [42]. Pitta et al. investigated the association between age and functional decline after TKA and suggested that there is a critical age at which functional decline begins regardless of the quality of the TKA procedure [43]. Although TKA is a successful surgery, it does not prevent ageing. Patient undergoing TKA may experience a decline in function with age. However, this functional decline does not necessarily indicate dissatisfaction.

The proportion of satisfied patients did not change substantially during the long-term follow-up period after TKA. In all five studies that had serial satisfaction data, more than 85% of the patients remained satisfied during the entire study period [23–25, 28, 32]. According to a systematic review of patient satisfaction after TKA by Kahlenberg et al., among the 138 studies that reported the percentage of satisfied patients, 82.6% reported greater than 80% satisfaction with the median percentage of 88.9% [16]. Although the follow-up periods were not mentioned in their study, their result was similar to ours. Other national registry data studies have reported similar rates. On the basis of the Swedish Knee Registry, Dunbar et al. reported that 17% of patients who underwent TKA were dissatisfied at the final follow-up [13]. Another national registry-based study by Baker et al. reported a satisfaction rate of 84% after TKA [44]. Our results confirmed that this level of patient satisfaction was maintained throughout the long-term follow-up. Although it is encouraging that the proportion of patients who were satisfied with their outcomes remained constant over the long-term period, a certain percentage of dissatisfied patients remained. The source of dissatisfaction after TKA varies and can be caused by factors other than medical reasons. Barrack et al. reported that socio-economic factors, specifically low household income, had a substantial impact on satisfaction after TKA [45]. In study by Bourne et al., patients who lived alone were more likely to be dissatisfied [46]. Surgeons need to better understand exactly what patients are dissatisfied with, and this requires a validated measurement tool. Since there are still no established systems for measuring patient satisfaction, further research is required [16].

This study has several limitations. Only a small number of studies were included in this review, and only a few reported sequential follow-up data on PROMs. Moreover, the quality of the included studies was low. Since our study aimed to report long-term follow-up results, many of the included studies had high dropout rates or were retrospective in design, leading to high heterogeneity. Despite these limitations, the importance of this study lies in demonstrating how PROMs and patient satisfaction change over time. This information can aid patients in making informed decisions regarding surgery. However, further controlled studies are required to address this topic.

## Conclusions

The overall PROMs after TKA were maintained, with improvement observed in the pain subscale until the mid-term follow-up. However, in the long-term, overall PROMs, including function and objective measure, declined compared with those in the mid-term. Despite the decline in the physical aspects of PROMs over the long-term follow-up period, the patient satisfaction rate remained consistently high throughout the study period. Providing this information to patient pre-operatively may assist in establishing realistic expectations.

## Supplementary Information

Additional file 1

Additional file 2

## Data Availability

The datasets used and/or analysed during the current study are available from the corresponding author on reasonable request.

## Abbreviations

TKA: Total knee arthroplasty
PROMs: Patient-reported outcome measures
MINORS: Methodological index for non-randomized studies
WOMAC: Western Ontario and Mcmaster universities osteoarthritis index
KSS: Knee society score
OKS: Oxford knee score
KOOS: Knee injury and osteoarthritis outcome score
KSCR: Knee society clinical rating
HSS: Hospital for special surgery
QoL: Quality of life
SF-12: 12-item Short Form survey
SF-36: 36-Item Short Form survey
SMD: Standardised mean difference
