# Supplementary material for: Serial changes in patient-reported outcome measures and satisfaction rate during long-term follow-up after total knee arthroplasty: a systematic review and meta-analysis
Source: Knee Surg Relat Res. 2024 Dec 4;36:43. doi: 10.1186/s43019-024-00241-6 (PMC11616191; doi:10.1186/s43019-024-00241-6)
Supplement: Supplementary file 1 — Additional file 1 [file 43019_2024_241_MOESM1_ESM.docx]

**Appendix 1** Electronic search strategy for each database

**MEDLINE**

1. (("arthroplasty, replacement, knee"[MeSH Terms] AND "long term*"[Title/Abstract] AND ("patient outcome assessment"[MeSH Terms] OR "patient satisfaction"[MeSH Terms] OR "clinical outcome*"[Title/Abstract] OR "functional outcome*"[Title/Abstract])) NOT "short*"[Title]) AND ((fft[Filter]) AND (english[Filter]) AND (1998:2023[pdat]))

**EMBASE**

1. ('patient-reported outcome'/exp OR 'patient-reported outcome' OR 'patient satisfaction'/exp OR 'patient satisfaction' OR 'quality of life'/exp OR 'quality of life' OR 'functional outcome'/exp OR 'functional outcome') AND ('total knee arthroplasty'/exp OR 'total knee arthroplasty') AND 'long-term' AND [english]/lim AND [1966-2023]/py

**SCOPUS**

1. TITLE-ABS-KEY ( ( 'patient-reported AND outcome' OR 'patient AND satisfaction' OR 'quality AND of AND life' OR 'functional AND outcome' ) AND 'total AND knee AND arthroplasty' AND 'long-term' ) AND PUBYEAR > 1998 AND PUBYEAR < 2024

**Cochrane Library**

1. ('patient-reported outcome' OR 'patient satisfaction' OR 'quality of life' OR 'functional outcome') AND 'total knee arthroplasty' AND 'long-term' in Title Abstract Keyword
2. Custom year range to 2023
