# Supplementary material for: Serial changes in patient-reported outcome measures and satisfaction rate during long-term follow-up after total knee arthroplasty: a systematic review and meta-analysis
Source: Knee Surg Relat Res. 2024 Dec 4;36:43. doi: 10.1186/s43019-024-00241-6 (PMC11616191; doi:10.1186/s43019-024-00241-6)
Supplement: Supplementary file 2 — Additional file 2 [file 43019_2024_241_MOESM2_ESM.docx]

Appendix 2 MINORS score of included studies

|  | Sebastia-Forcada, 2023 | Wylde, 2021 | Baek, 2021 | Woo, 2021 | Bajada, 2019 | Scott, 2019 | Arikupurathu, 2019 | Jiang, 2017 | Wiliams, 2013 | Arthur, 2013 | Meding, 2012 | Watanabe, 2004 | Schrøder, 2001 |
| --- | --- | --- | --- | --- | --- | --- | --- | --- | --- | --- | --- | --- | --- |
| 1. A clearly stated aim | 2 | 2 | 2 | 2 | 2 | 2 | 1 | 2 | 2 | 2 | 2 | 1 | 1 |
| 2. Inclusion of consecutive patients | 2 | 2 | 2 | 2 | 2 | 2 | 2 | 2 | 2 | 2 | 2 | 2 | 2 |
| 3. Prospective collection of data | 1 | 2 | 1 | 1 | 1 | 2 | 1 | 2 | 2 | 2 | 1 | 2 | 2 |
| 4. Endpoints appropriate to the aim of the study | 2 | 1 | 1 | 2 | 1 | 1 | 1 | 2 | 2 | 1 | 2 | 1 | 2 |
| 5. Unbiased assessment of the study endpoint | 2 | 0 | 0 | 2 | 2 | 2 | 2 | 1 | 0 | 2 | 1 | 0 | 0 |
| 6. Follow-up period appropriate to the aim of the study | 2 | 2 | 2 | 2 | 2 | 2 | 2 | 2 | 2 | 2 | 2 | 2 | 2 |
| 7. Loss to follow-up less than 5% | 2 | 1 | 2 | 2 | 2 | 1 | 2 | 1 | 1 | 1 | 2 | 1 | 1 |
| 8. Prospective calculation of the study size | 0 | 0 | 0 | 2 | 0 | 0 | 0 | 0 | 0 | 0 | 0 | 0 | 0 |
| TOTAL MINORS score | 13 | 10 | 10 | 15 | 12 | 12 | 11 | 12 | 11 | 12 | 12 | 9 | 10 |
| Maximum possible score | 16 | 16 | 16 | 16 | 16 | 16 | 16 | 16 | 16 | 16 | 16 | 16 | 16 |
